# Exercise-Based Pain Interventions for People with Dementia Using a Biopsychosocial-Environmental (BPSE) Model

**DOI:** 10.1007/s13670-025-00450-1

**Published:** 2025-11-10

**Authors:** Annalisa Na, Joke Bradt, G. Peter Gliebus, Julie A. Fritz, Laura Gitlin

**Affiliations:** 1 Drexel University, Philadelphia, 60 N. 36th Street, PA 19104 USA; 2 Marcus Neuroscience Institute, Baptist Health, Boca Raton, FL USA; 3 University of Utah, Salt Lake City, UT USA

**Keywords:** Pain, Dementia, Exercise, Aging, Mobility, Comorbidity

## Abstract

**Purpose of review:**

To introduce the Biopsychosocial-Environmental (BPSE) model to guide an exercise intervention to address pain related to osteoarthritis (OA) among people living with dementia (PLWD). This review [1] synthesizes literature on the biological, psychological, social, and environmental aspects of pain from OA in the knee, neurodegeneration in PLWD, and exercise implications for these comorbidities, and [2] demonstrates the clinical utility of the BPSE model in designing tailored exercise interventions to manage knee OA pain in PLWD.

**Recent findings:**

Research indicates that pain from knee OA and neurodegeneration from dementia interact across multiple factors, including psychological, social, and environmental. These factors compound the biological interactions of the comorbidities, further challenging function and quality of life.

**Summary:**

The BPSE model provides a structured framework for tailoring exercise interventions to PLWDs’ interests and preserved abilities. Applying this model may improve pain management, mobility, and quality of life while informing future chronic disease management in PLWD.

## Introduction

Chronic pain, or pain persisting beyond the expected healing period (≥ 3 months), is a leading cause of functional decline among older adults (≥ 65 years), impacting over 50% of community-dwelling people living with dementia (PLWD) [1, 2]. Knee osteoarthritis accounts for >65% of chronic pain conditions in PLWD and limits weight-bearing tolerance, leading to mobility decline, falls, challenging behaviors, poor quality of life, and premature mortality [3–5]. As the aging population grows, the burden of pain and dementia is expected to rise, exacerbating functional decline, increasing healthcare costs, and straining caregivers [5, 6]. These trends highlight a need for effective, sustainable interventions for chronic pain management in PLWD.

Exercise, structured and purposeful physical activity, is an evidence-based nonpharmacological intervention that supports physical, functional, and emotional well-being in older adults with chronic pain and those with dementia [7–10]. However, most pain management programs reported in the literature for PLWD include passive approaches, lack pain mechanism specificity, and have been shown to be ineffective [11]. To date, exercise has not been systematically used to address chronic pain in PLWD [12]. This review introduces the Biopsychosocial-Environmental (BPSE) Model, a novel framework to guide the examination and design of a tailored exercise program for knee OA pain management in PLWD.

Purpose of this review is to (1) synthesize literature on the biological, psychological, social, and environmental aspects of pain relating to knee OA, neurodegeneration relating to ADRD, and exercise implications for the comorbidities and (2) demonstrate the clinical utility of the BPSE Model to design tailored exercise interventions to manage knee OA pain in community-dwelling PLWD.

## BPSE Factors Relating to Pain and Neurodegeneration

### The BPSE Model Offers a Comprehensive, Person-Centered Framework for Assessment and Intervention Design

**Fig. 1:**
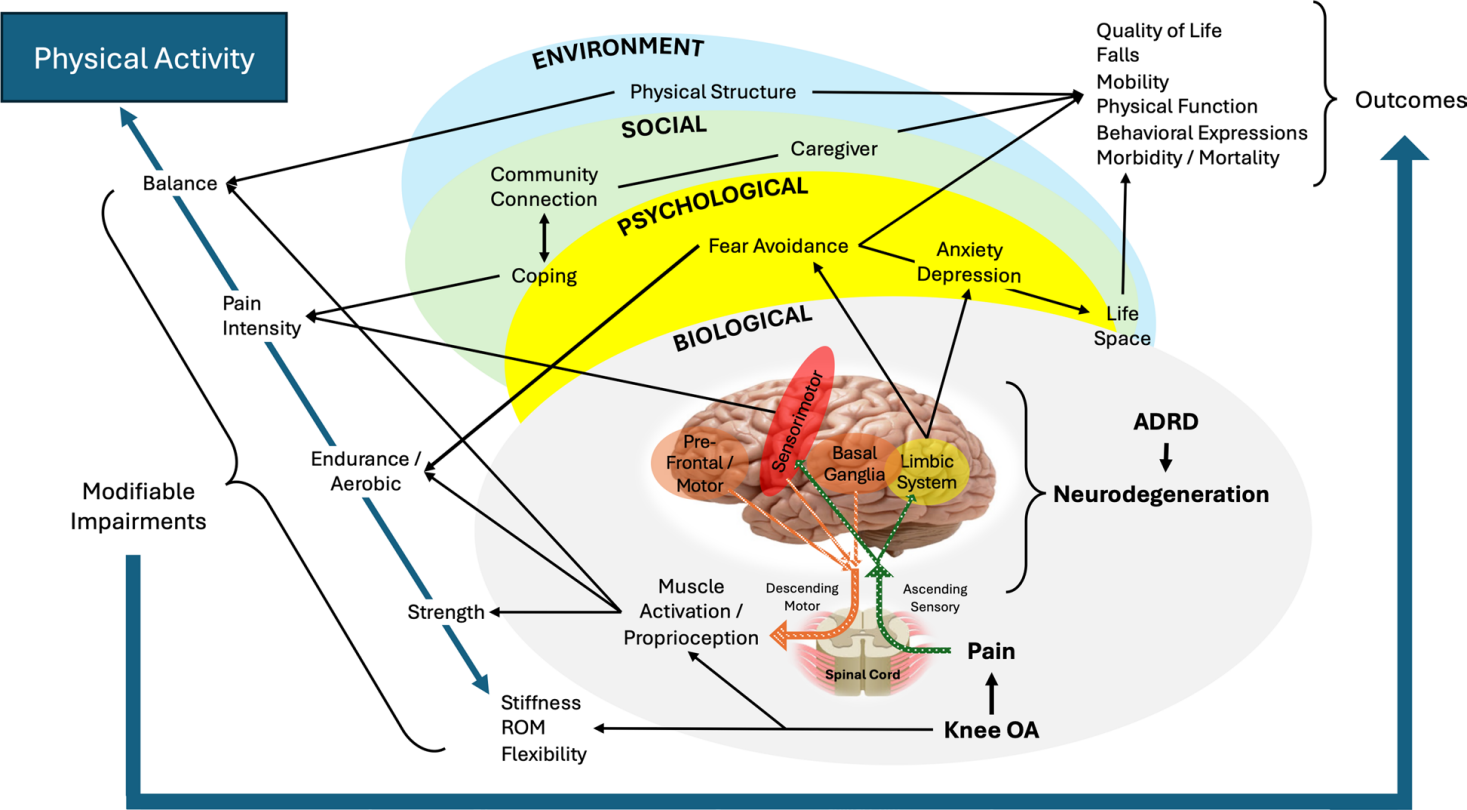
The BioPsychoSocial-Environmental (BPSE) Modelillustrating ($\rightarrow$) interconnections across biological, psychological, social, and environmental domains between pain processing (knee osteoarthritis [OA]) and neurodegeneration (Alzheimer’s Disease and Related Dementias [ADRD]). Modifiable impairments from the domains can be targeted with physical activity interventions to improve outcomes such as quality of life. ($\rightarrow$) Peripherally ascending sensory pathway; ($\rightarrow$) Centrally descending motor pathways; ($\rightarrow$) Address through physical activity intervention

Its design is guided by existing literature and models on biological, psychological, social, and environmental factors relating to pain, dementia, and at their intersection. For this review, we primarily focus on Alzheimer’s disease; however, for clarity and consistency with cited sources, we use “dementia” and ADRD interchangeably to encompass Alzheimer’s disease, mild cognitive impairment (MCI), other dementia types where noted, and adopt the term PLWD when referring to this population. The interconnections across the domains for ADRD and knee OA pain in community-dwelling older adults are summarized in Fig. 1.

### Biological Interplay Between Pain and ADRD

Pain signals, originating from peripheral nociceptors, such as those elicited by knee OA, ascend the spinal cord to cortical and subcortical regions. These pain networks, intertwined with the neurodegeneration of ADRD, impact pain processing and motor inhibition.

 ***Pain Processing*** occurs via lateral (sensory-discriminative) and medial (affective-emotional) pathways that are impacted by ADRD differently. The sensory-discriminative component is responsible for pain detection, sensation, and discrimination, and is processed in the somatosensory cortex, regions impacted in late-stage ADRD [13, 14]. The affective-emotional components are responsible for pain interpretation and emotional response, such as linking pain to fear, anxiety, and threat learning. This process occurs in the anterior cingulate cortex, dorsolateral prefrontal cortex, and anterior insula of the limbic system [15], which is impacted by early ADRD [16]. Hence, early ADRD affects emotional responses to pain, leaving pain sensation (detection and intensity) intact [16, 17]. The variability in how dementia subtypes and stages impact pain processing is beyond the scope of this review, though important to acknowledge and is reviewed elsewhere [18].

***Motor inhibition*** related to pain and ADRD arises from the brain’s sensory, motor, and executive control networks, as well as downstream effects from muscles and movement systems, leading to altered movement patterns and walking difficulty [19–21]. Localized to the affected joint, degeneration caused by OA triggers inflammatory and pain responses that impair motor activation [22] and proprioception [23]. Peripheral pain nociceptors are processed centrally in the anterior cingulate cortex of the limbic system, supplementary motor area of the motor cortex, and basal ganglia. Pain-causing persistent nociception activation causes maladaptation, leading to central sensitization and increased motor and sensory circuit excitability, further impacting muscle activation. Neurodegeneration from ADRD impacting the limbic and prefrontal cortex further challenges the motor pathways, disrupting movement initiation and the ability to adapt to environmental challenges. There are recent suggestions that ADRD may be impacting mobility (e.g., shorter strides and slower walking speeds) before cognitive symptoms are detectable [24]. Though preliminary, and the mechanisms remain unclear, this may suggest pre-clinical implications of ADRD on motor control. The combined effects of OA pain and ADRD deficits can compound sensorimotor integration, disrupting the integration of proprioceptive, visual, and vestibular input, which in turn compromises balance, increasing risk for falls, accelerating functional decline, and reducing quality of life [24–26].

### Psychological Interplay Influencing Pain Experiences in PLWD

OA pain elevates the risk of depression and anxiety in PLWD, further intensifying disability and contributing to poor outcomes [27–29]. Pain in the presence of depression and anxiety often promotes fear-avoidant beliefs and maladaptive coping that accelerate functional decline. Learned fear in relation to pain can lead to activity avoidance, withdrawal, and deconditioning [30].

In ADRD, impaired memory, diminished cognitive function, and altered emotional regulation limit the ability to interpret, communicate, and cope with pain. Executive cognitive function and semantic memory shape how pain is perceived, interpreted, and anticipated [31]. Consequently, PLWD who already face heightened risks of mobility loss and social isolation [32] may be especially susceptible to catastrophizing, activity avoidance, and accelerated functional loss, though these relationships remain underexplored in this population.

#### Social Factors Influencing Pain in PLWD

Relationships among PLWD, their caregivers, and communities can influence pain experiences and quality of life. Social connections are associated with emotional regulation, pain perception, and coping ability. Kitwood’s Person-Centered Care Model emphasizes that supportive environments for PLWD preserve autonomy and cognitive function, whereas isolation and disengagement exacerbate decline and behavioral changes [33]. However, strategies leveraging social connections for pain management in PLWD are lacking.

***Caregivers***, formal (paid) or informal (unpaid family members or friends), play a central role in supporting PLWD. Among community-dwelling PLWD, caregivers serve as primary observers of status changes, including behaviors that may indicate pain. However, caregivers’ stress and health literacy abilities can impair accurate behavioral interpretation, leading to under-recognition or misattribution of pain and inadequate management [34, 35]. A recent study suggests that training caregivers to identify and assess pain in individuals with ADRD can enhance their confidence in recognizing and communicating pain [36].

***Culture and community*** can shape how individuals perceive illness and engage in care, including beliefs about aging, pain, ADRD, and exercise. Viewing pain as an expected part of aging may delay care-seeking, while norms around emotional expression, gender, and health literacy can suppress symptom reporting. Cultural beliefs shape perceptions of exercise, including risks and appropriateness. Factors such as distrust in the healthcare system, language barriers, traditional healing preferences, and family dynamics can influence treatment decisions.

#### Environmental Considerations Relating To OA Pain in PLWD

The environment encompasses the physical spaces where PLWD reside (e.g., private home) and receive care (e.g., clinics). While examined in ADRD and aging research, environmental factors are less explored in pain research. Perhaps insights from ADRD care can inform strategies to mitigate environmental triggers that impact pain and mobility. Cohen-Mansfield’s Stimulation Balance Model describes how high- and low-stimulation environments can worsen agitation and associated behaviors [37]. Gitlin and Hodgson’s Socioecological Model [38], building on Lewin and Bronfenbrenner [39], frames care within a multi-level environment ranging from individual to policy levels [40]. These frameworks highlight the need for adaptable, co-constructed environments that support the individual. Modifications such as simplifying spaces can encourage mobility by reducing pain triggers. Meanwhile, environmental factors such as incorporating sensory and visual stimuli can distract the individual from their pain or serve as cues to exercise. Auditory cues, such as alarms or music, can remind or even motivate a PLWD to exercise. Other environmental cues can facilitate embedding exercise engagement into daily routines, like exercising during commercial breaks when watching television or toe-walking down a specific hallway.

***Life space*** is the total environment in which a person lives and interacts [38]. Pain-avoidant behaviors in older adults are associated with shrinking life space [41]. Considerations for life space crosses BPSE domains being impacted by modifiable impairments and influencing outcomes. (Fig. 1).

#### Exercise Prescription

The BPSE model provides a framework to guide exercise programs in the specific management of knee OA impairments in PLWD. General exercise guidelines for older adults recommend ≥ 150 min/week of moderate-intensity aerobic activity, muscle-strengthening ≥ 2 days/week, and regular balance and flexibility training [42, 43]. Training parameter recommendations can vary based on body composition and should be considered [44]. The following exercise dosing is reported for OA pain management [7, 45–48] or among PLWD [10, 49].

***Exercise intensities*** are defined by heart rate (HR) (i.e., % of HR max [HRmax] or reserve [HRR]), rate of perceived exertion (RPE), or descriptors per the Talk Test [50], and are used to define exercise parameters below. RPE is based on the Borg Scale, ranging from 6 (Light) to 20 (Maximal Exertion) [51] or its modified version, ranging from 0 (No exertion) to 10 (Absolute maximum effort) [52]. For aerobic, balance, and flexibility training, recommended intensities range from light to moderate: [42, 44] *Light* = 57–63% of HRmax; RPE is 9–10 (Borg) or 2 (of 10 on modified); or the ability to talk and sing comfortably during exercise. *Moderate* = 64–76% of HRmax; RPE is 12–13 (Borg) or 3–4 (of 10 on modified); or the ability to talk but not sing during exercise [44].

Many prevalent health conditions (e.g., cardiac, pulmonary, or neurologic conditions) and medications (e.g., beta-blockers or certain antidepressants) can cause atypical HR responses, warranting exercise intensity to be determined with RPE or the Talk Test. For PLWD, cognitive or severe communication impairments may limit the ability to self-report or multitask (e.g., completing the aerobic exercise, self-assessing, and self-reporting perceived exertion), warranting exercise intensity to be determined using HR. Careful monitoring is necessary to ensure dosing for therapeutic effectiveness, while also minimizing risks, including overexertion, as older adults with ADRD or OA may have reduced physiological capacity due to sedentary lifestyles.

***Aerobic exercise*** involves continuous movement of large muscle groups, elevating heart and respiratory rates for sustained periods (e.g., walking, cycling, swimming, and dancing), improving cardiovascular efficiency, endurance, and mobility [49]. Aerobic training dosing parameters include 20–60 min sessions, 3–7 days per week, at moderate intensity [44]. In PLWD, aerobic exercise, alone or paired with cognitive training, improves brain function [53, 54]. For OA, aerobic exercise stimulates endorphin release and joint fluid mobilization beneficial for pain, stiffness, and mobility [47, 55]. However, weight-bearing aerobic activities (e.g., biking, walking) can be challenging for severe or irritable lower extremity OA symptoms. Lower-impact activities for joint protection, limiting repetitive loads, and improving tolerance may be warranted [42]. Studies show that low-impact, moderate-intensity aerobic exercises can achieve benefits comparable to vigorous exercises [56, 57].

***Balance exercises*** are structured activities designed to improve postural control by improving stability, neuromuscular coordination between the nervous system and muscles, postural response strategies, reaction time, and cognitive-motor integration (e.g., dual-task), thereby supporting functional mobility and reducing fall risk [58, 59]. Activities can include static (e.g., single-leg stance) or dynamic postural training (e.g., walking or Tai Chi). Balance dosing parameters include 1–7 times per week at light intensity, with 1–2 sets of 4–10 exercises [44]. In PLWD, dual-task training, introducing cognitively challenging tasks with balance activities, improves executive function, attention, and mobility [58, 60]. Balance training, similar to aerobic, often includes weight-bearing activities that may exacerbate OA symptoms and require adaptations. Recent studies on OA-related modifications of Tai-Chi routines yielded effective outcomes, including among PLWD [61–63].

***Flexibility exercises*** involve muscle lengthening or movements to maintain or restore joint ROM, reducing stiffness, and facilitating mobility [45, 48]. It can also promote relaxation, provide sensory stimulation, and support mood and engagement. Recommended dosing includes 60-second holds, repeated 2–3 times per joint or muscle group [64]. These light-intensity activities can be performed daily, incorporated into routines, and performed actively, with assistance, or passively by a caregiver or clinician, depending on the individual’s capacity and needs. In OA, ROM limited by bony structures (e.g., osteophytes) may not be amenable to such conservative interventions. Forceful stretching is likely to cause pain and is unlikely to improve motion.

***Resistance exercises*** entail short-duration activities performed against external resistance (e.g., free weights, machines, resistance bands, body weight) to enhance muscle strength, maximal aerobic capacity, and mobility. In older adults, resistance training supports muscle mass, bone density, metabolic health, and cognitive function. These physiological benefits are essential for preserving physical function and reducing the risks of multimorbid, age-related decline common to those with ADRD, including sarcopenia and frailty. In those with mild cognitive impairment or early dementia, resistance training is effective for improving gait, balance, and mobility. Targeted strength training directed at OA is effective at redistributing mechanical loads from affected joints to muscles, improving pain and physical function. Recommended dosing is 2–3 days per week, performing 1–3 sets of 8–12 repetitions targeting 8–10 muscle groups at moderate to high intensity (70–80% of one-repetition maximum [1RM] for strength; 40–60% of 1RM for power). Loads should induce fatigue without causing pain. For example, adjusting painful mid-range knee extension resistive exercises (65°–45°) to a more tolerable range (30°–0°).

Exercise parameters must align with individual needs through ongoing assessment, with adjustments guided by performance, symptoms, and person-centered goals. Implementation, regardless of ADRD severity, should be supervised and routinely reassessed. Exercise can also benefit from being paired with multimodal approaches, including modalities (e.g., electrical stimulation, heat/cold), analgesic medication, and cognitive and behavioral coping strategies (e.g., distraction, pacing, relaxation techniques, or music) to help tolerate pain and exercise programs.

#### Examination and Assessment Across BPSE Domains Exercise Programs for Knee OA Pain in PLWD

Designing an exercise program targeting OA pain for PLWD requires a comprehensive assessment by clinicians across the BPSE domains. Program design and delivery must be tailored to the preserved cognitive and physical abilities, while recognizing the diverse and evolving needs of the individual, such as preferred communication strategies and key impairments limiting physical function. Many of the validated measurements frequently used in clinical practice by healthcare providers require self-reporting, problem-solving, or following directions, which may necessitate family, friends, or caregiver involvement. Table 1 summarizes key assessments per the BPSE domains for knee OA pain in PLWD.

**Table 1:** Assessments components guided by the Biopsychosocial-Environmental (BPSE) model to design an exercise intervention

Domain	Level	Assessment	Program Implications
Biological	PLWD	• Medical history and medication • Pain (mechanism, location, severity, duration, irritability, what makes it better/worse) and other symptoms (e.g., stiffness, instability)	• Precautions or contraindications related to exercise (e.g., blood pressure, heart rate, or rate of perceived exertion) • Differentiating symptoms as related to pain versus dementia.
		• Strength, range of motion, endurance • Physical function, health, independence (e.g., ADLs / IADLs), mobility, transfers, and balance. SPPB, Grip Strength, Timed Up and Go (Dual-task), Stair Climb Test, 6 min Walk Test • Movement patterns – avoidance behaviors, movement patterns	• Exercise dosing defined by physical impairment, function, and movement patterns associated with pain. • Indicators for intensity - progress, sustain, or regress
		• Cognitive function (Dementia Type, Severity, cognitive status, executive function, other cognitive domains) • Communication ability and preferences	• Sequencing and design (e.g., problem solving, cueing, repetition): Number of activity steps • Goal and outcomes • Education delivery / strategies
Psychological	PLWD	• Depression, anxiety, fear, fear of pain, mood • Perception of self, memory changes, and life changes • Beliefs, attitudes, or experience(s) regarding pain, exercise, dementia, and/or aging	• Activity Selection Consideration: Perceived meaning, interesting, or useful to PLWD; Challenging, do-able, and yields observable gains • Education and support: Pain coping strategies and ADRD
Social	PLWD	• Habits, preferences • Cultural Values / Native Language • Family, friends, support group • Personhood (e.g., support team and relationships)	• Strategies to build into daily routine • Who to involve (caregiver, family, friends) • Insurance: number of sessions approved • Transportation
	Caregiver	• Who is involved in care, how many people, availability, and roles and relationships • Living Situation • Caregiver’s medical history, medications, physical function, education, prior experience, and burden/mental health • Attitudes toward pain, dementia, exercise, physical activity, and general health literacy • Willingness to assume the caregiving role and level of resistance or acceptance • Access to resources relating to mental health care and other supports	• Extent and ability to involve the caregiver • Consistency of external support that may require remote or flexible strategies • May need training in skill-building for caregiver • Education and reframing perceptions regarding pain, dementia, or exercise
Environment	Residential Space	• Life spaces and related stressors (physical, emotional, cognitive) • Housing layout (multi-level vs. single, steps/stairs, handrails, distance between rooms, flooring type, shower setup/safety bars, proximity to bathrooms) • Setting: urban, suburban, or rural • Safety and security (wandering risk, neighborhood safety, alertness): access to safe areas for walking and activities • Availability of transportation • Persons sharing the living space • Health and wellness resources • Environmental prompts to support exercise	• Home modifications, adaptive equipment, or treatment priorities • Implications of environmental cues, monitoring, or supportive technologies • Simplify routines and reduce clutter may support • Education regarding resources, exercise opportunities, and health and wellness strategies • Use of prompts and supports (calendars, reminders, structured cues) for appointments and exercise programs
	Place of care delivery	• Staff and personnel competencies • Facility accessibility (entrances, hallways, restrooms, spaces) • Safety features of equipment • Availability of quiet, low-stimulation spaces	• Staff training in dementia care and exercise delivery • Appointment systems for consistent or flexible scheduling, timely reminders
	All Physical Space Consideration	• Lighting: ability to adjust amount and type • Flooring: non-glare surfaces, avoidance of high-contrast dark/light patterns, safe transitions between floor types • Sensory stimulation: o Visual: clutter, number of people, lighting consistency o Auditory: ambient noise, echoes, background sounds Oversight needs: Time of day, task or activity specifics, supervision versus the amount of assistance	• Home care assessments to identify environmental barriers, daily routines, and opportunities to integrate exercise into familiar contexts • Lighting adjustments to reduce confusion, agitation, or visual discomfort • Flooring modifications (non-glare, uniform surfaces, removal of distracting patterns or hazards) can reduce fall risk and improve confidence in mobility. • Sensory stimulation that balances between over and under stimulation • Levels of supervision and cueing

#### Exercise Risks

Chronic comorbid conditions beyond OA and ADRD can dictate exercise precautions, contraindications (e.g., cardiovascular disease), and define training boundaries before initiating programs. Symptoms such as dizziness, nausea, fatigue, muscle weakness, and breathlessness can indicate exercise intolerance, prompting further medical attention. It is also worth noting that while adherence to contraindications and precautions remains essential, age-related biases frequently result in subtherapeutic exercise prescriptions. Existing literature demonstrates that prescribing intensities sufficient to elicit therapeutic adaptations yields benefits for older adults, including those with chronic disease and PLWD [53, 65].

#### Physical examination

Examining and assessing symptoms, physical impairments (strength, endurance, balance), and functional mobility are necessary for determining program priorities and key modifiable impairments. Integrated with subjective reports, examination findings can confirm or refute clinical hypotheses to determine whether the impairments associated with functional limitations are appropriate to modify with exercise interventions. (Fig. 1). At the intersection of OA pain and ADRD, the impact of cognitive abilities on measurement selection and interpretation needs to be considered.

***Pain intensity*** relies on self-report, commonly using standardized measures including the Numeric Rating Scale. Consequently, pain is often underreported or misinterpreted in PLWD due to impaired verbal communication, altered pain expression, and fluctuating cognitive status. A multimodal approach, including self-report, proxy-report, observations, and validated behavioral pain assessment tools (e.g., Pain Assessment Checklist with Limited Ability to Communicate [PACSLAC] or Pain Assessment in Advanced Dementia [PAINAD]), is recommended. These tools assess behavioral cues as potential indicators of pain status and intensity (e.g., facial expressions, vocalizations, changes in activity or social interaction). Limitations to using these tools would be differentiating whether behaviors are related to pain or other unmet needs (e.g., anxiety, hunger). Psychometric quality of these tools is reported in recent reviews [66–68].

***Physical performance tests*** to assess functional mobility, balance, and strength (e.g., Timed Up and Go [TUG], grip strength) associated with or caused by OA pain. Excellent to good reliability is reported for most performance tests when used among individuals with mild to moderate ADRD, but additional research is required for severe ADRD [69]. Reassessing these measurements at regular intervals supports monitoring disease progression, adaptation to interventions, and maintenance of safety.

#### Program Priority

Identifying how OA pain impacts mobility and functional goals in PLWD is essential for determining program progress, effectiveness, and outcomes. Assessment across the BPSE domains should help capture the unique intersection between comorbid OA pain and ADRD for each individual, thereby guiding the development of a person-centered program that prioritizes functional goals. These goals should be collaboratively and iteratively developed with the PLWD and their caregivers to ensure the program is meaningful and reflects personal values, preferences, and daily routines. Goals should be simple, relevant, and achievable within the modifiable and non-modifiable boundaries of the PLWD’s cognitive and physical abilities. Incorporating caregiver input can help align exercise priorities with safety, autonomy, and quality of life, while regular review and adjustment can accommodate cognitive or functional changes as needed. Emphasizing program priorities based on functional goals (e.g., increasing participation in community events, attending family outings, or improving functional independence) can facilitate engagement outcomes.

### Facilitating Engagement of PLWD in an exercise-based Pain Program

Without consistent and meaningful participation of PLWD in exercises, effectiveness would likely remain limited. The BPSE framework, as described, provides a structured and comprehensive approach to inform program design. Which, when combined with the 3-Step Tailored Approach, can facilitate active engagement even as ADRD evolves. Key factors identified across the domains are integrated into the 3-Step Tailored Activity Approach, so that it includes in step 1-tailoring to the individual, step 2-optimizing the environment, and step 3-incorporating caregiver involvement when appropriate [40]. (Table 2) This integrated approach facilitates activity selection and program design based on the individual’s interests and cognitive and physical abilities so that exercises are meaningful and appropriate, facilitating engagement and effective outcomes.

**Table 2:**
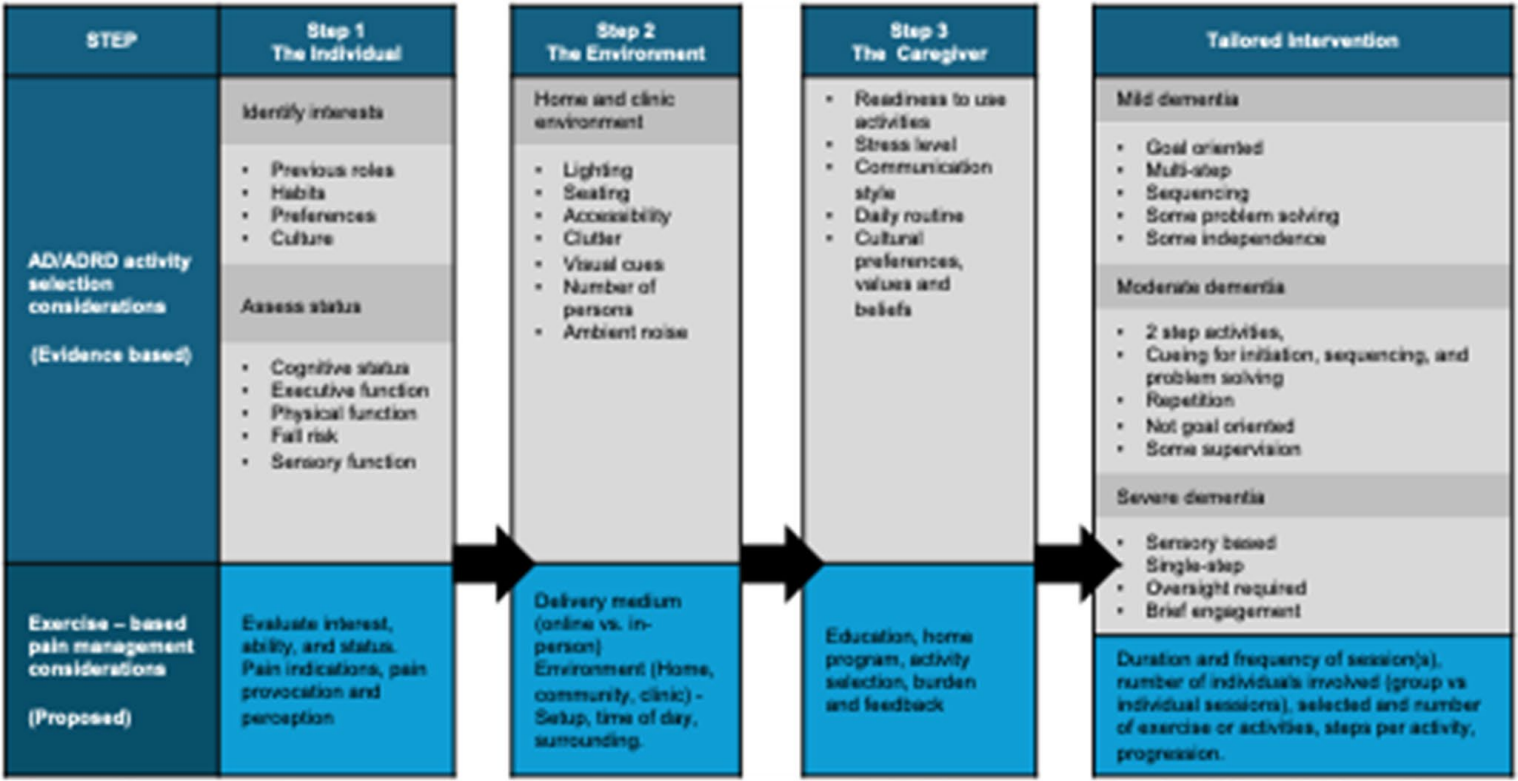
Integrating the BPSE model based on a 3-step tailored approach [40] to tailor activities and facilitate engagement of an exercise-based knee osteoarthritis pain intervention program for community-dwelling older adults with varying dementia severity

***Tailoring to the individual*** involves building an exercise program based on the PLWD’s interests and abilities. Meaningful and familiar activities can evoke procedural memory, leveraging intuitive movement to enhance participation. For example, gardening movements may appeal to a retired horticulturist, while mimicking caregiving tasks might resonate with a former nurse. Delivery approaches (e.g., communication strategies, language, and framing) tailored to the individual’s perceptions can build trust. For example, a former dancer may respond more positively to “dancing” than “exercise” and engage better when mirroring activities instead of following verbal instructions. Dosing strategies for targeted training, such as strength or endurance, could be progressed through music selection of varying tempo and duration or by adding ankle weights.

***Optimizing the environment*** can include addressing barriers and promoting facilitators to enhance program engagement and success. Effective environmental modification can enhance autonomy, support mobility, enhance orientation, and implement routines that match the PLWD’s energy levels and schedule. Whether in the home or clinic, environments should support exercise goals, mitigate risks, facilitate sensory regulation, and establish routines. In the home, adaptations can include structural modifications such as adding handrails to stairs or a grab bar next to hard-to-open doors, the toilet, or the bathtubs. Chairs can be added to long hallways to serve as a rest stop for walking routines or a visual cue to complete sit-to-stand exercises for functional strength training. Both in the clinic and the PLWD’s home, clutter reduction is important to decrease fall risk, facilitate mobility exercises, and minimize overstimulation. The process to optimize the environment may require personalized checklists to assess structural elements (e.g., flooring, lighting, assistive furniture) and sensory stimuli (e.g., noise, temperature, visual distractions).

***Incorporating caregiver involvement*** can be imperative to program success and outcomes. Caregivers who are familiar with the PLWD’s needs, health status, and preferences can provide motivation, supervision, cueing, emotional support, and navigate communication barriers. Involving caregivers in goal-setting and program adaptation can foster collaborative care and be beneficial for negotiating strategies to integrate activities into daily life and enhance outcomes. However, the extent and type of caregiver involvement are highly variable and dependent on a variety of factors (Table 2. Step 3).

The BPSE framework is useful for guiding the shared and targeted components across domains to identify key modifiable impairments with precise exercise dosing for PLWD to achieve their functional goals. When paired with the 3-Step Tailored Approach, activities selected and adapted to the evolving needs of the PLWD are integrated into existing evidence-based knee OA pain exercise programs.

### Strategies, Challenges, and Future Directions

Chronic pain related to knee OA provides an important starting point for introducing the BPSE model, given its high prevalence and impact on mobility in PLWD. Existing evidence-based guidelines for knee OA management recommend exercise interventions targeting periarticular muscles at therapeutic doses for addressing aerobic capacity, strength, balance, and range of motion (ROM) impairments [7, 8, 45, 48, 55]. These recommendations, however, are based on studies that explicitly exclude PLWD. Moreover, many of the exercises require executive cognitive function and multi-step task performance. To facilitate engagement among PLWD, these exercises need to be adapted and tailored to the preserved physical and cognitive abilities and interests of the individual. Such strategies include using functionally meaningful activities and delivering them within supportive environments. Deviating from traditional exercise while maintaining OA-specific therapeutic doses preserves the efficacy while enhancing participation by aligning with the PLWD’s preferences and needs.

The BPSE model addresses complex and intersecting challenges in managing knee OA pain among PLWD. Despite its potential, the model has not yet been operationalized or tested. Future research should evaluate the feasibility, adaptability, and effectiveness of BPSE-informed interventions, particularly in diverse settings. Next, preconceived biases among healthcare professionals and caregivers as an important challenge many older adults face. Particularly, older adults with OA or ADRD cannot benefit from or participate in exercise, undermining established benefits and often resulting in no exercise or underdosing. Although the BPSE model does not explicitly address clinician or caregiver biases, it promotes individualized, person-centered intervention design that may help counter generalized assumptions. Further, as with any exercise-based intervention, strategies for prolonging the achievements gained are often challenging for any population, but especially for PLWD and in the context of knee OA pain. Though sustaining exercise behaviors is beyond the scope of this review, we highlight a few considerations unique to this population. For example, setting realistic, functional, and meaningful goals. Goals that facilitate independent mobility (e.g., stair climbing) or participation in community events (e.g., grandchild’s graduation) can foster autonomy, self-efficacy, and motivation. Community-based programs, such as ADRD-friendly fitness classes or walking groups, can provide structure, routine, and social connection while enhancing continued engagement in exercise. With intentional planning and support, exercise can be a powerful, long-term tool for preserving function, autonomy, and well-being in this population.

Currently, the BPSE model is one of the first to provide structured guidance for designing interventions that address the complex interplay of biological, psychological, social, and environmental factors in chronic comorbid conditions, such as knee OA pain and ADRD. The BPSE model builds on established biopsychosocial models and descriptions [70] such as the International Classification of Functioning, Disability and Health [71]. However, the BPSE Model distinguishes the environment as its own domain rather than subsuming it under social. This distinction highlights the unique impact of physical environments (e.g., home safety, sensory stimuli, accessibility) alongside biological, psychological, and social domains (e.g., caregiver dynamics, cultural beliefs). The model recognizes sociocultural and physical environments as distinct determinants shaping health outcomes, aligning with the National Institute on Aging [72–74], the National Institute on Minority Health and Health Disparities [75], and the 3-Step Tailored Approach for dementia care [40].

By guiding the successful implementation of effective, tailored exercise interventions for knee OA pain, the BPSE model may support the adaptation of evidence-based interventions for managing other chronic conditions common among PLWD. Conditions such as cardiovascular disease, diabetes, and frailty present similar challenges, including multimorbidity, functional decline, environmental barriers, and a need for person-centered exercise programs that can be guided by the BPSE model. Assessing the BPSE model’s applicability across these conditions and care contexts is critical for determining its broader utility as a comprehensive framework for delivering individualized, person-centered care in dementia populations. Additional future considerations can also include incorporating technology, such as wearables, telehealth platforms, and virtual reality, as avenues to enhance personalization, monitor engagement, and expand access. Their integration, together with interdisciplinary collaboration, can help embed exercise into routine ADRD care and strengthen chronic disease management strategies.

## Conclusion

Comorbid OA and ADRD present a convergence of pain, psychosocial vulnerabilities, and environmental challenges. Although exercise is known to benefit both conditions independently, few interventions address them concurrently or holistically. The BPSE model bridges this gap by guiding the development of tailored, multidomain exercise programs that account for individual goals, contextual factors, and caregiver dynamics. This review illustrates how the model supports comprehensive assessment and person-centered care. It demonstrates the importance of integrating caregivers, modifying home and clinical environments, and aligning interventions with meaningful activities to improve adherence and quality of life.

## Key References

- Na A, Buchanan TS. Self-reported walking difficulty influences gait characteristics in patients with medial compartment knee osteoarthritis. *Clin Biomech (Bristol)*. 2022;100:105805.
  - **Important** – This study investigates the association between self-perceived walking difficulty, a concept rooted in the psychological domain, and gait biomechanics, which are mechanistic and biological factors modifiable through exercise. This study is an important key reference because its findings, combined with recommendations from clinical practice guidelines, provide valuable exercise-based treatment implications for knee OA pain described in this review.
- Na A, Sefcik JS, Gitlin LN. Nonpharmacological Pain Management for People With Dementia: A Scoping Review Mapping Research Gaps From a Pragmatic Lens. J Am Geriatr Soc. 2025.
  - **Very Important** – This scoping review provides an up-to-date summary of the existing literature on pain management in dementia care, illustrating a lack of exercise-based interventions applicable to the heterogeneous characteristics of PLWD (i.e., variability across cognition and residency environments). This study is a very important key reference for this review, as it demonstrates the need for feasible and acceptable exercise-based intervention designs for community-dwelling older adults with varying dementia severity. Findings from this study, combined with established guideline recommendations, highlight discrepancies and disparities in the management of common chronic conditions that need to be addressed and improved in dementia care.
- Xiao Y, Fan Y, Feng Z. A meta-analysis of the efficacy of physical exercise interventions on activities of daily living in patients with Alzheimer’s disease. *Front Public Health*. 2024;12:1485807.
  - **Very Important** – This meta-analysis describes program dosing and delivery parameters for engaging PLWD in exercise programs that yield cognitive and physical function benefits. This study is a very important key reference for this review because it demonstrates the feasibility, acceptability, and tolerance of PLWD with varying severity of ADRD to engage in exercise programs at effective doses. Program design and delivery strategies can help inform the development of exercise-based programs for pain management in PLWD.
- Vassilaki M, Aakre JA, Lesnick TG, Kremers WK, Graff-Radford J, Knopman DS, et al. Patterns of Factors in the National Institute on Aging Health Disparities Research Framework Domains and Mild Cognitive Impairment Risk. *AJPM Focus*. 2025;4(3):100324.
  - **Important** – This study demonstrates how biological, psychological, social, and environmental factors shape cognitive impairment risk among older adults with different backgrounds. This study is an important key reference for this review because it describes the importance of integrating contextual considerations into intervention planning for PLWD, thereby highlighting the need to distinguish and evaluate environmental and social factors both independently and across other domains.

## Data Availability

No datasets were generated or analysed during the current study.
